# How do Co-agents Actively Regulate their Collective Behavior States?

**DOI:** 10.3389/fpsyg.2016.01732

**Published:** 2016-11-04

**Authors:** Jérôme Bourbousson, Marina Fortes-Bourbousson

**Affiliations:** “Movement, Interactions, Performance” Laboratory (EA4334), University of NantesNantes, France

**Keywords:** interpersonal co-ordination, local couplings, co-regulation, social systems, joint action, team coordination, complex adaptive system

## Introduction

Collective capability of producing patterned collective behaviors is one important field of research in work psychology (e.g., shared cognition approach, Fiore and Salas, 2004; interactive team cognition approach, Cooke et al., 2013), neurosciences (e.g., social neuromarkers, Tognoli et al., in press; neurological mirroring, Waldman et al., 2015), sociology (Miller, 2013), or human movement science (e.g., joint movement, Schmidt and Richardson, 2008; team behavior, Araujo and Bourbousson, 2016). Within this stream of research, one neglected topic has been to conceptualize how interactors regulate online their dynamic involvement in collective activity, which is the individual skillful activity of adjusting online to the needs of the collective behavior. Grounded in of the hypothesis that collective behavior emerges from a self-organized complex system, the present opinion discusses the nature of the active regulation of the interactions performed by the co-agents. A deeper grasp of this regulation process is needed to understand how and why interpersonal co-ordination forms, stabilizes and/or is destroyed, leading to the emergence of high order phenomena at the team scale that are not fully predictable from the individual activities that compose the social system under study.

Collective behavior is deemed here to constitute the property of a social system composed of living entities. In research that has considered collective behavior as emerging from a self-organized complex system, an important focus has been on the between-agents' interactions, supported by an informational flow that binds agents (e.g., Schmidt et al., 1990). In this stream of research, information is defined as an ambient energy that disturbs the agent, depending on his current activity (Varela et al., 1991). From the (interpersonal) informational flow, individual activities can be entrained, mutually affected by others' movements, so that the emerging collective behavior cannot be conceived out of either the nature or the content (i.e., being non-representational) of such a flow (e.g., Kelso, 1994; Lagarde and Kelso, 2006; Richardson et al., 2007). However, while between-agents informational flow has been considered the main binding mechanism that makes collective behavior emerge, we aim at pointing out that the way individuals manage their interaction in the real-time mainly has been theoretically presupposed rather than empirically investigated. We will use empirical and logical evidence to highlight shortcomings in the actual theorizations of the way individual movements merge into a collective unit. In our opinion, current research should restrict the importance of the *co-regulation* and the *local couplings* hypotheses. Both hypotheses appear unsatisfactory to us, and might probably be refined through a further consideration of the social system's size effects as a main topic.

## Hypothesis 1: a collective behavior emerges from individual activities being locally coupled

According to the unifying principle of non-linear dynamical systems (see Jirsa and Kelso, 2013 for further detail on the co-ordination dynamics approach,), the collective behavior of a complex system emerges as the result of self-organization among the interacting individual parts that comprise the system, such as humans in a social system (see Schmidt and Richardson, 2008, for details on interpersonal co-ordination research). Thus, considering social systems as the place where collective behaviors emerge leads to the assumption that global collective patterns observable at the social system level of organization come from indivisible interpersonal dynamical couplings at a lower level of organization, also called *local couplings*. In this light, the rhythm of the collective behavior is supposed to change intermittently between periods of stable and unstable behaviors, depending on the capability of interacting parts to maintain or change their local coupling with respect to the evolving environment in which the social system is embedded (Glassman, 1973).

In such a conceptualization, interplays between the high (i.e., global) and low (i.e., local) levels of organization have been of particular interest (Rio and Warren, 2016). The emergence principle accounts for the process by which local couplings give rise to a higher order identifiable pattern, the so-called collective behavior. The global pattern that emerges thus cannot be reduced to the sum of its individual components, and cannot be predicted by the sole properties of these components. Conversely, the downward causation principle accounts for the process by which the global patterned behavior constrains the way in which individual agents behave and interact at the local level of organization, without these agents necessarily being aware of such a descending causality. According to the principle of parsimony of scientific explanations, and largely inspired by swarming intelligence theorizations, this *local couplings* hypothesis has been very successful in explaining from simple mechanisms how complex social systems behaviors can emerge from simple local rules of interaction.

## Hypothesis 2: emergent collective behaviors are supported by a process of ≪ co-regulation ≫ at the level of the local couplings

At a local level of organization, what allows a social system to exhibit the signatures of complex systems and thus let emerge a dynamical collective behavior? One important contribution that synthetized theoretical answers to this question came from the enactivist theory of interpersonal couplings (e.g., De Jaegher and Di Paolo, 2007). As the starting point, a collective behavior is captured through the identification of non-accidental patterns of individual behaviors, as observed at the global scale. These patterns can be captured by various tools, such as those well-developed for spatiotemporal pattern identification (Gudmundsson and Horton, 2016). However, an identifiable patterned behavioral co-ordination is not enough to consider that a collective behavior has emerged from interaction of its constituent individual parts; it also is required that the given interactors actively regulate the interpersonal co-ordination dynamics at the level of their local couplings. In other words, an informational flow must have occurred between them, and this flow must be dynamically managed. In a more fundamental way, De Jaegher and Di Paolo (2007) stated that complex phenomena of emergence are facilitated when both interactors simultaneously regulate their ongoing interpersonal co-ordination (i.e., a bi-directional flow of interplay), making the collective behavior achieved escape from any individual perspective of the interactors implied. In this specific case, the collective behavior can express all the marks of complexity and meta-stability needed to consider the social system as exhibiting self-sustained dynamical behaviors. The need for such a mutuality in the interaction fit under the theme of *co-regulation* requirement, also discussed as a *mutual awareness* requirement in other research traditions (Fiore and Salas, 2004).

Some studies revealed the crucial function of this co-regulation requirement in interpersonal interactions, especially in those that used the perceptual crossing paradigm (Auvray et al., 2009). This device puts two actors in situations where they have to move an avatar in a virtual environment populated by different entities (avatars of humans and various lures), visually empty but providing tactile stimulation at each encounter through the mouse used by the participants. Interestingly, what helps participants to succeed in finding each other, and subsequently to experience social connectedness, is the occurring co-regulation process they both perceived simultaneously at some instances (Froese et al., 2014a,b), regardless of the extent to which each actor was satisfied by the unfolding interaction, since they were not informed of their current effectiveness in the task. In agreement with the co-regulation requirement for interpersonal co-ordination emergence, most of the studies testing this regulation process have been experimental and have focused on the co-ordination within dyads, providing reiterated evidence of the interpersonal benefits related to co-regulation processes (Schmidt and Richardson, 2008).

## Perplexing empirical evidence 1: social systems do not need co-regulation to perform

While the hypothesis of a co-regulation requirement has been pervasive in interpersonal co-ordination research, some empirical studies have found it hard to observe in naturalistic empirical data, especially in goal-directed collective behaviors. For instance, Bourbousson and colleagues investigated how agents heeded their co-agents in the study of basketball teams performing in their natural social competitive context (Bourbousson et al., 2015). The authors examined mutual adjustments at the level of the activity that was meaningful for the interactors, and compared novice and expert teams. Teams were considered dynamic social networks, with team members as nodes and members' awareness of other members during ongoing performance as relations. Networks, and changes to them across games, were analyzed at different levels of organization, using social network analysis to identify patterns of co-regulation within the teams. Notably, the results showed that the reciprocity index, accounting for the instantaneous co-regulation occurring within all the considered dyads within the teams, was significantly lower than expected by chance when considering expert team co-ordination, but was not the case in novice team co-ordination. Moreover, the observed low co-regulation was very stable over time, so that the proposed intra-team patterns of regulation had all the marks of expertise. Other studies have reported similar observations in various field of team co-ordination, as in civilian command, control, and communication settings (Wellens and Ergener, 1988), socio-technical collaborative systems (Salmon et al., 2008), or various settings of cognitive engineering research (Cooke et al., 2009). Together, these studies suggested an enhanced capability of expert social systems to achieve and maintain an optimal level of awareness during the unfolding activity, with this level of awareness being lower than in novice social systems.

In this light, it appeared reasonable to the authors to consider that interactors' activities of regulation directed toward co-agents become parsimonious through practice and expertise enhancement, possibly enabled by a gradual establishment of implicit co-ordination processes (Bourbousson et al., 2015). Implicit co-ordination processes mean that interactors co-ordinate by drawing on accurate expectations of future intra-team events. These expectations are developed and shared by interactors through extensive shared practice prior to their current activity (Eccles, 2010; Gorman, 2014). It appears that whatever the nature of the process involved, expert interactors probably do not need to pay as much attention to their co-agents during ongoing task performance, as a result of their shared experiences. The co-regulation hypothesis is thus quite unsatisfactory, at least as a strong interaction requirement in goal-directed social systems that are composed of many inter-related dyads, and in which the shared experience of interactors allows them to adopt a parsimonious but effective structure of regulation of the intra-team co-ordination.

## Perplexing empirical evidence 2: human agents can grasp the global picture they help to make emerge

As introduced above, a main inspiration to collective behavior understanding has come from swarm intelligence, as observed in social insects (Theraulaz, 2014). Collective behaviors of social insects are powerful forms of collective intelligence because local couplings have been shown to be sufficient to give rise to very patterned and adaptive collective behaviors. Most of the time, agents do not even need to be strictly coupled together, as long as each of them maintains its coupling to the shared environment. Most complex-systems-inspired frameworks of interpersonal co-ordination have thus subsequently considered that local couplings were enough to conceptualize collective behaviors, that these local couplings signed a parsimonious way of structuring informational flows within the social system, and that such a process was a perfect example of the emergence phenomenon. However, unlike the research on social insects, that on interpersonal co-ordination has neglected to consider that human co-agents are capable of grasping the global picture they help to make emerge, especially in cases in which collective behavior is goal-directed and actively regulated by co-agents. In this way, the collective behavior in which individuals are involved may directly support their adaptive activity and thus be considered as a non-negligible informational constraint that supports humans' goal-directed behavior. This capability has been called *holoptism*, that is the ability for any interacting co-agent to perceive the dynamics of the whole interactive system (Noubel, 2004; Bauwens, 2005).

For instance, sport coaches are well aware of such a capability for holoptism in humans: When players are called to perceive the rhythm of the game, free spaces, or team fluidity of movements, the given agents thus couple to high-order spatiotemporal information that probably helps them to better couple locally^1^, but this information does not rely *per se* at the local coupling level itself (see Bourbousson et al., 2014 for an empirical research). Out of the sports domain, similar observations have also been discussed in the field of designing collaborative digital tools. For instance, Bauwens (2005) suggested looking with caution at swarming intelligence systems, and proposed that the peer-to-peer process might be re-considered in light of the quality of holoptism that is offered to user experience through digital collaborative practice.

While the local couplings hypothesis is very useful in swarming behaviors theories, our opinion is that current interpersonal co-ordination theories in humans run the risk of not being cautious enough when introducing the local couplings hypothesis as a starting point of the research (e.g., Silva et al., 2014). One can note that most of the experimental study designs have invited participants to adjust to a single co-agent, but this individual dyad level of investigation does not clearly distinguish local and global scales of the collective behavior (e.g., Schmidt and Richardson, 2008): When participants are asked to co-ordinate their arms in a dyad, by locally coupling with the movement of the co-agent, they also directly regulate the global co-ordination dynamics to which both are contributing, so that local and global perceptual capabilities coincide in the task goal. Thus, our opinion is that one approach to further investigate what holoptism may bring to interpersonal co-ordination theories might be to extend the number of co-agents implied in the collective behavior under study to better allow for the distinction between the levels of organization that shape the social system's dynamics. For instance, such an extension of the number of participants involved in the study design would allow for discussing human capability of switching their attention from local couplings to the global interpersonal pattern.

## Breaking the deadlock: considering that the number of co-agents matters in theorizing social systems functioning

Where does the problem probably lie? First, we have to remember that very few studies investigated how people actively are involved in regulating their interpersonal co-ordination states in the real-time. When this active regulation was discussed in the research, it was often considered a theoretical assumption related to the nature of the informational flow binding actors, rather than being empirically investigated and described. From this starting point, we have challenged two theoretical hypotheses, the co-regulation and the local couplings hypotheses, respectively. Our opinion is that both have been overlooked, probably due to a common property of the existing study designs: The number of co-agents implied in the experimental paradigms was quite small (i.e., two interacting agents; Alderisio et al., 2016). Studying dyads may have limited our fundamental understanding of how collective behaviors emerge from interacting individual activities. Empirical and theoretical benefits should thus come from studying operating social system larger than a dyad, especially by revising the co-regulation and the local couplings hypotheses.

What does it change to consider the number of co-agents implied in the study design as a variable? In the literature, few studies show how the number of agents involved in a given collective behavior really matter and can change the processes needed to make a collective behavior effective and adaptive. For instance, the effect of the co-agents' number has been studied abundantly in social insects' science, and is known as the effect of size colony on the adjustment processes. To illustrate, Perna et al. (2012) investigated termite colonies and identified two main adjustment processes that may explain the emergence of collective behaviors. The first process is a purely local mechanism that accounts for an arrangement of agents' behaviors based on only local information. The second process is a local estimation of global properties, and accounts for agents being sensitive to the efficiency of the current collective behavior (i.e., through rudimentary sensory sensitivity) and of improving on it based on information about some global parameters of the existing social system (Perna et al., 2012). Interestingly, the given insects were shown to be probably capable of switching from the first to the second process when the social system exceeded a threshold in term of colony size–the first process being less resilient to environmental changes or unpredictable events.

Obviously, the topic of co-agents' number was not discussed enough in human behavior science, but a few examples may be found in numerical science, especially in human crowd modeling, that explain how human collective systems can exhibit adjustment mechanisms that change, and are very dependent on the number of co-agents (Mehran et al., 2009). Some examples can also be found in the study of financial market fluctuations where interactions between agents are considered a variable (e.g., Lux and Marchesi, 1999), but these interactions are not expressed as a linear function of the investors' number but rather as subjected to a threshold effect that makes social contagion more or less pronounced (e.g., Orléan, 1990). Specificity of human collective behaviors often relies on interpersonal co-ordination being itself the goal to achieve, implying that co-agents interact to actively create/maintain/disrupt global interpersonal states of behavior, and, in some instances, these states are probably managed through holoptism capability. Empirical studies that investigate effects of the social system's size on the collective behavior of humans who are actively regulating their online states of co-ordination will contribute to an opened avenue of research on the topic of interpersonal co-ordination dynamics. Unanswered questions thus would need to be addressed, like knowing how many members implied in the social system might require or prevent occurrences of holoptism or one-sided co-ordination processes.

## Perspectives

How can informational flows be patterned in goal-directed social systems larger than dyads? For instance, in the case of co-agents reciprocally co-regulating their activities in a 5-member social system, each interactor must regulate four co-ordination links at once, which makes the attentional requirement of the task very hard to manage, and even harder in a 10-member social system in which 45 co-ordination links have to be simultaneously co-regulated, and so on. To counter-balance the co-regulation hypothesis, it is probable that co-regulation can occur only between certain co-agents, and the overall social system functions through few co-ordination links (i.e., low density within the network of informational flows). It is also likely that the coupling linkages do not necessarily need to be reciprocal between the co-agents, so that one-sided co-ordination should provide benefits to the global efficiency and parsimony of the system. It is even more likely that co-agents can face the difficulty of regulating each local coupling by grasping the overall picture at some point in their activity (i.e., global matching capabilities), thus counter-balancing the local couplings hypothesis. Related questions should then be addressed: does structural congruence between members, as achieved through recurrent interactions in team training (Maturana and Varela, 1987), help them to pay less (reciprocal) attention to the regulation of their couplings? Do some properties of interpersonal networks allow for a lessened need of agents' co-regulation, due to a somewhat ‘less effort for more effects’ phenomenon, such as might be hypothesized in wide networks? To which extent does holoptism capability help to better explain the emergence of non-goal-directed (i.e., unintentional) patterns of collective behavior? These proposals need to be challenged through empirical data analysis in future research, which should allow better theorization of how co-agents couple through skillful dynamic individual adjustments.

## Author contributions

JB originated the questioning. JB, MF co-wrote the manuscript.

### Conflict of interest statement

The authors declare that the research was conducted in the absence of any commercial or financial relationships that could be construed as a potential conflict of interest.

## Footnotes

^1^The question remains open whether holoptism only apply to goal-directed collective behavior, or may also be implied in spontaneous motor entrainment (i.e., unintentional interpersonal coordination patterns emergence).
